# Endothelial Progenitor Cells in Diabetic Retinopathy

**DOI:** 10.3389/fendo.2014.00044

**Published:** 2014-04-09

**Authors:** Noemi Lois, Rachel V. McCarter, Christina O’Neill, Reinhold J. Medina, Alan W. Stitt

**Affiliations:** ^1^Centre for Experimental Medicine, School of Medicine, Dentistry and Biomedical Science, Queen’s University Belfast, Royal Victoria Hospital, Belfast, UK

**Keywords:** diabetic retinopathy, diabetic macular edema, endothelial progenitor cells, ischemia, proliferative diabetic retinopathy, retina, hematopoietic stem cells, vessels

## Abstract

Diabetic retinopathy (DR) is a leading cause of visual impairment worldwide. Patients with DR may irreversibly lose sight as a result of the development of diabetic macular edema (DME) and/or proliferative diabetic retinopathy (PDR); retinal blood vessel dysfunction and degeneration plays an essential role in their pathogenesis. Although new treatments have been recently introduced for DME, including intravitreal vascular endothelial growth factor inhibitors (anti-VEGFs) and steroids, a high proportion of patients (~40–50%) do not respond to these therapies. Furthermore, for people with PDR, laser photocoagulation remains a mainstay therapy despite this being an inherently destructive procedure. Endothelial progenitor cells (EPCs) are a low-frequency population of circulating cells known to be recruited to sites of vessel damage and tissue ischemia where they promote vascular healing and re-perfusion. A growing body of evidence suggests that the number and function of EPCs are altered in patients with varying degrees of diabetes duration, metabolic control, and in the presence or absence of DR. Although there are no clear-cut outcomes from these clinical studies, there is mounting evidence that some EPC sub-types may be involved in the pathogenesis of DR and may also serve as biomarkers for disease progression and stratification. Moreover, some EPC sub-types have considerable potential as therapeutic modalities for DME and PDR in the context of cell therapy. This study presents basic clinical concepts of DR and combines this with a general insight on EPCs and their relation to future directions in understanding and treating this important diabetic complication.

## Diabetic Retinopathy

### A worldwide disease with increasing prevalence

Diabetic retinopathy (DR) is the most common microvascular complication of diabetes mellitus (DM) and a leading cause of visual loss among individuals of working age (1, 2). Due to the ever-increasing numbers of people with DM, principally the type 2 form of disease, it is expected that the burden of DR will continue to rise. Indeed, it has been estimated that the worldwide prevalence of DR will increase from 126.6 million in 2010 to 191 million by 2030 (3).

Diabetic retinopathy occurs more frequently in people with poor glycemic control and with longer duration of diabetes (4). Other major risk factors include hypertension (5), renal disease (6, 7), and dyslipoproteinemia (8, 9). The Diabetes Control and Complications Trial (DCCT) (10) and the United Kingdom Prospective Diabetes Study (UK-PDS) (5) demonstrated the importance of keeping a tight glucose control on delaying the onset and slowing the progression of DR in people with type 1 and type 2 DM, respectively. The UK-PDS demonstrated that maintaining adequate levels of blood pressure (~140/80) reduced the risk of DR in people with DM type 2 (5). Although HbA_1c_, an index of prolonged hyperglycemia, remains the strongest risk factor for predicting progression of DR, this parameter accounted for only 11% of the risk of retinopathy in the DCCT (11). Furthermore, HbA_1c_, blood pressure, and total serum cholesterol, together, accounted for only 9–10% of the risk of DR in the Wisconsin epidemiologic study of diabetic retinopathy (WESDR) (12). The situation continues to become ever-more complex, and recent evidence suggests that sleep apnea (13, 14) and changes in serum prolactin (15), adiponectin (16), and homocysteine (17) may affect the progression of DR.

### Clinical features and classification

The earliest retinal abnormalities detected in people with DM appear to be functional in that they occur in a fundus, which appears normal. Functional changes in the diabetic retina include abnormal electroretinographic responses (18, 19), changes in blood flow, and loss of autoregulatory mechanisms that adjust retinal capillary perfusion (20). As DR progresses, microaneurysms, retinal hemorrhages, hard exudates (lipid leakage), and “cotton wool spots” (localized disruption of axoplasmic flow) may develop, which are readily observed upon fundus examination. In late stages of DR, venous beading (irregularity in the caliber of the retinal veins with saccular dilations and thinning of the vein wall), intra-retinal microvascular abnormalities (IRMA), and intra-retinal and/or pre-retinal neovascularization will ensue.

**Figure 1:**
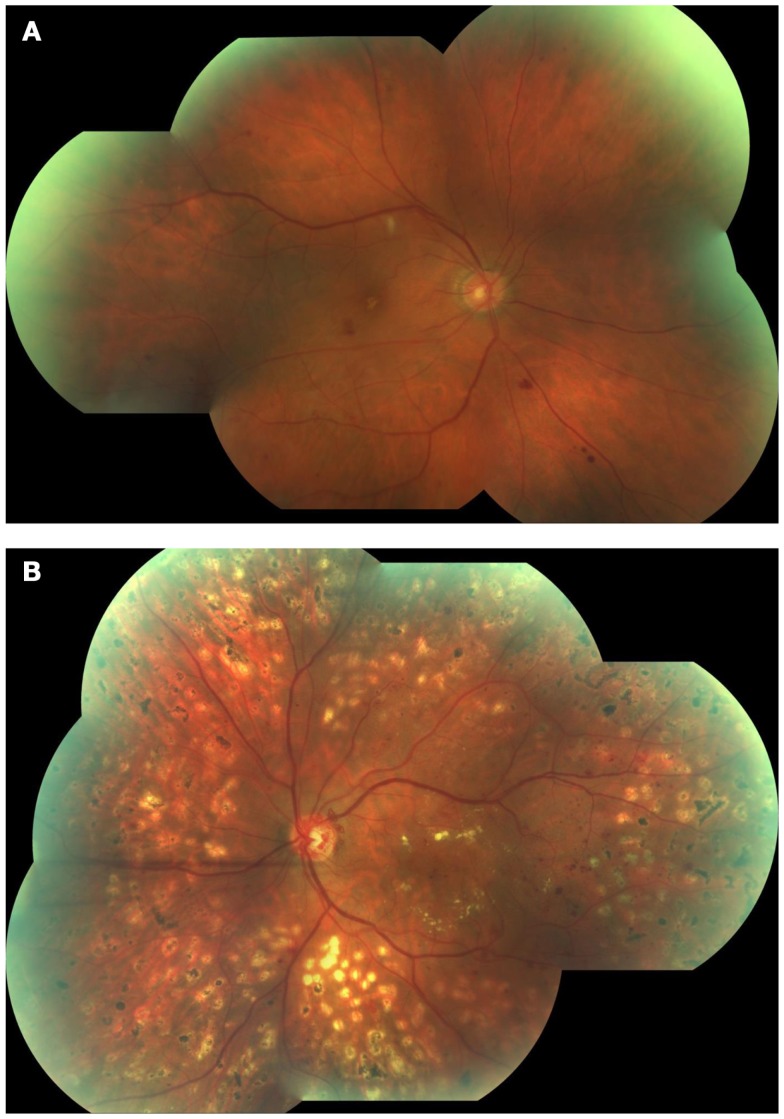
**Fundus photographs obtained from patients with non-proliferative diabetic retinopathy (NPDR) (A) and proliferative diabetic retinopathy (PDR) (B)**. Note few hemorrhages and a cotton wool spot at the superior aspect of the macula in **(A)**, and marked neovessels in the disc and macular exudation in **(B)**.

**Figure 2:**
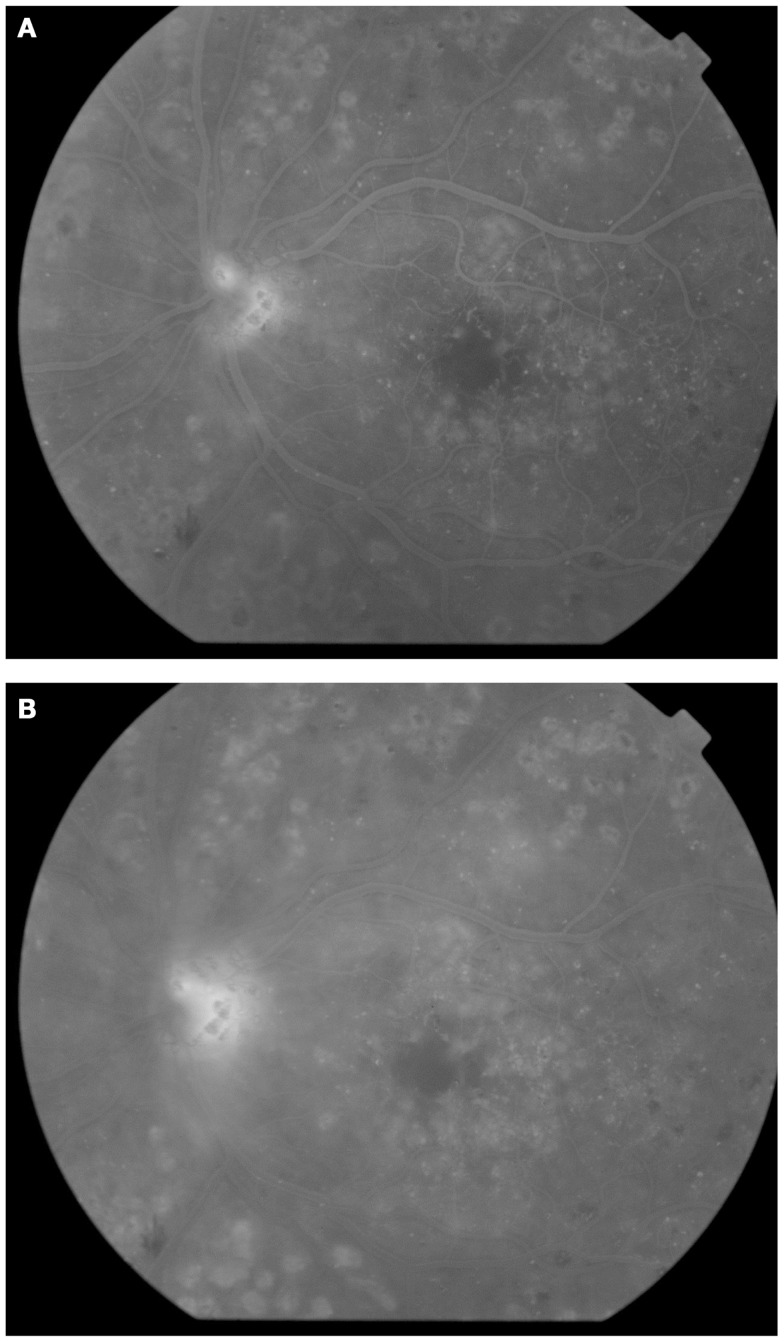
**Fluorescein angiography obtained from the left eye of a patient with diabetic macular edema and PDR**. Note drop out of perifoveal capillaries **(A)** with late leakage **(B)** and leakage from neovessels at the disc**(A,B)**.

Based on the absence or presence of neovascularization, patients with DR are classified as having non-proliferative diabetic retinopathy (NPDR) or proliferative diabetic retinopathy (PDR), respectively (Figure 1). The fundus of patients with severe NPDR are typically characterized by the presence of retinal hemorrhage and/or microaneurysms in four retinal quadrants, venous beading in two retinal quadrants, or IRMA in one retinal quadrant (known as the “4–2–1 rule”). These patients are at higher risk of progression to PDR. At NPDR or PDR stages, there may be overt breakdown of the inner and/or outer blood retinal barrier (BRBs) with characteristic diabetic macular edema (DME) (Figure 2). The accumulation of fluid at the center of the retina occurring in DME constitutes the leading cause of visual loss among people with DR.

### Vascular abnormalities

The pathological sequence of retinal vessel abnormalities in DR includes early and progressive thickening of the basement membrane and dysfunction and loss of endothelial cells, pericytes, and vascular smooth muscle [reviewed by Curtis et al. (21)]. Progressive capillary non-perfusion and ensuing ischemia in the diabetic retina may induce hypoxia-related up-regulation of cytokines and growth factors, such as vascular endothelial growth factor (VEGF) (22, 23), and this drives vasopermeability (DME) and/or abnormal neovascularization (21).

The diabetic milieu is highly damaging to the retinal capillary endothelium and this results in a significantly greater turnover of these cells when compared to non-diabetic equivalents (24). Exhaustion of endothelial cell replicative capacity and cells reaching their so-called Hayflick limit (25) means that the retinal vasculature in diabetic patients has impaired regenerative capacity. This underlines the progressive degenerative nature of DR in most patients.

### Retinal ischemia and diabetic retinopathy

Retinal ischemia is involved in the occurrence of the two major complications of DR, namely DME and PDR. Surprisingly, little is known about the natural history of retinal ischemia in DR and how it is modified by treatment. Ischemia can affect different areas of the retina; its implications with regard to the development of complications of DR may depend on this topographic distribution and also on its extension. For example, a relatively small area of ischemia affecting the perifoveal capillaries (“ischemic maculopathy”) may have profound impact on vision. In contrast, a large area of midperipheral retinal ischemia may not produce an immediate change in sight but may trigger a neovascular response with development of PDR and poor longer term outcomes. In certain circumstances, very anterior (far peripheral) retinal ischemia, even if limited, may give rise to anterior segment neovascularization (rubeosis), including the development of neovascular glaucoma, and a more guarded prognosis. It is not clear why in some patients areas of retinal ischemia are mainly restricted to the macula, whereas in others the retinal vasculature is relatively intact at the macula but marked midperipheral retinal ischemia develops.

**Figure 3:**
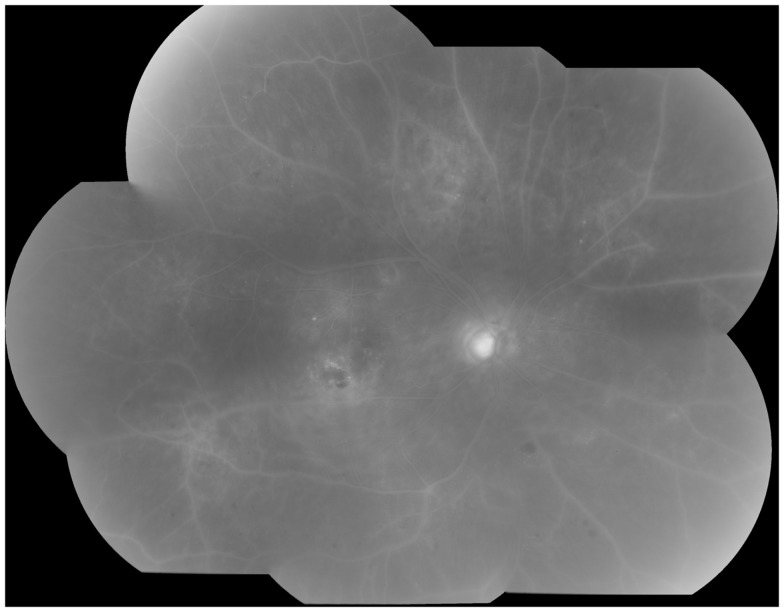
**Fluorescein angiography obtained from the right eye of a patient with non-proliferative diabetic retinopathy**. Note diffuse retinal non-perfusion and ischemia in the midperipheral retina in the absence of neovascularization.

**Figure 4:**
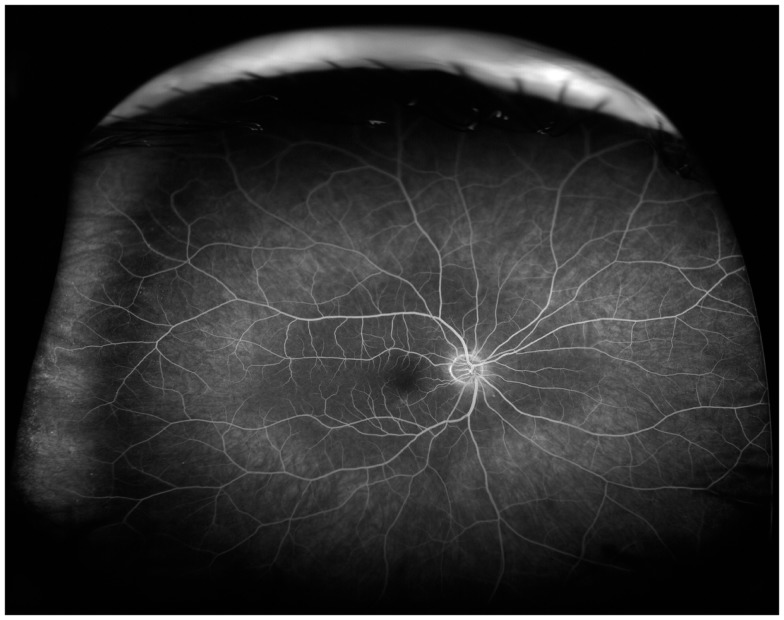
**Wide-angle fluorescein angiography obtained with the Optos imaging system**. Note excellent visualization of the vascular tree in the posterior pole and midperipheral retina with a single short.

Clinically, retinal ischemia can be adequately identified, and its extension measured, by means of fundus fluorescein angiography (FFA) (Figure 3). For this imaging technique, a dye (fluorescein) is injected in a peripheral vein; images of the fundus are obtained as the dye circulates through the retinal blood vessels. Recently, new emerging technologies using wide-angle fundus cameras have become available (26, 27). These allow obtaining angiograms encompassing a 200-degree field of the retina in a single shot with a nearly complete visualization of the whole retinal vascular tree (Figure 4). Other new imaging techniques are being developed, such as differential phase-contrast swept-source optical coherence tomography (OCT), which allows visualization of retinal blood vessels without the need for injection of dyes (28–30).

**Figure 5:**
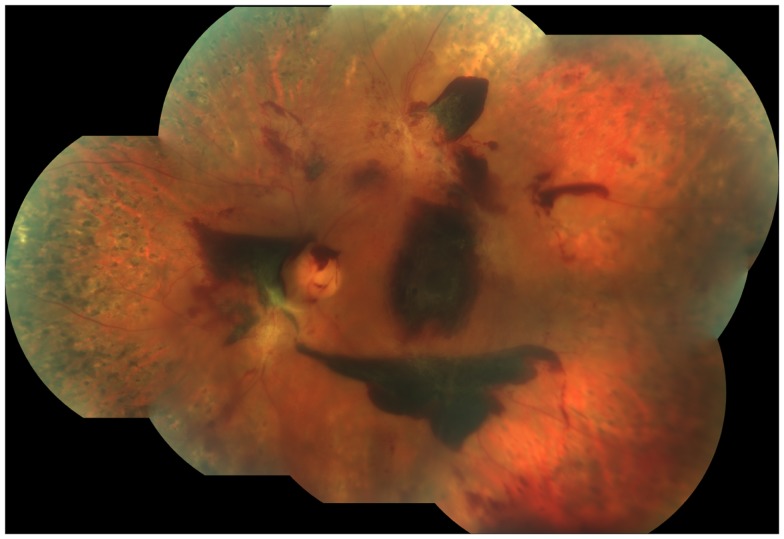
**Fundus photograph obtained from the left eye of a patient with proliferative diabetic retinopathy who had undergone laser panretinal photocoagulation (PRP)**. Despite a complete PRP, active disease was still present, as demonstrated by the presence of still viable neovascularization and extensive sub-hyaloid, pre-retinal hemorrhages. Note the large pre-retinal hemorrhage covering the fovea.

Using FFA, spontaneous resolution of areas of retinal ischemia in DR has been observed (31), although the mechanisms underpinning re-perfusion have not been elucidated. Currently, no treatment has been identified that can reverse retinal ischemia in DR. For example, the current treatment for PDR, laser panretinal photocoagulation (PRP), aims at destroying the ischemic retina rather than at reperfusing it. Furthermore, progression of the disease can still be observed following PRP (Figure 5). As discussed below, circulating EPCs may play a role in vascular re-perfusion and tissue regeneration. In addition, delivery of these cells has emerged as an exciting potential therapeutic strategy for retinal ischemia in DR.

## Endothelial Progenitor Cells

Endothelial progenitor cells (EPCs) are circulating cells believed to play an important role in tissue regeneration by promoting the repair of blood vessels and aiding in the re-perfusion of ischemic areas (32). EPCs generally account for only ~0.01% of circulating cells, although reported levels varied which may, once again, reflect on the variability on the cells measured (33). Their origin remains somewhat uncertain; they may derive from hematopoietic stem cells in the bone marrow and/or highly specialized vascular stem cell niches in vessel walls or within the endothelium (34, 35). Controversy also exists with regard to the definition of an EPC. Pre-clinical and clinical investigations evaluating the therapeutic potential of EPCs have produced variable results, with some studies showing favorable outcomes whereas others failed to demonstrate success (36–39). It is likely that this variable response observed in EPC therapy relates, at least in part, to the use of a heterogeneous mixture of cells (not just EPCs) rather than being explained by the lack of therapeutic potential of a well-defined, efficacious EPC sub-type.

EPCs have been proposed as useful cell biomarkers of disease (40). For both therapeutic and biomarker investigations to be successful, it is essential that well-defined populations of EPCs are used. It would be advantageous if agreement among researchers in the EPC field could be reached, especially with regard to the definition and terminology used to refer these cells and their various sub-types. If the same type of EPCs were to be used for therapeutic or prognostic purposes by researchers, comparisons among studies and pooling of their data, required to increase the power of the evidence, would be possible, furthering the research into this exciting area. As discussed below, unfortunately, this has not been the case so far with regard to the existing literature on EPCs in DR.

### Defining an EPC

Groundbreaking research by Asahara et al. (32) first isolated endothelial cell progenitors from human peripheral blood using CD34 and Flk-1 (VEGF receptor 2, VEGF-R2) surface markers. They determined that these cells were able to differentiate into endothelial cells and incorporate into sites where active angiogenesis was taking place. Subsequent studies by other groups provided supporting evidence on the existence of circulating EPCs (41–44).

As pointed above, controversy exists with regard to how to define an EPC. In a very recent article, Basile and Yoder thoroughly reviewed this issue and provided a useful perspective on the characterization of EPCs (45). They suggested that evidence support the concept that endothelial colony-forming cells (ECFC), also known as outgrowth endothelial cells (OECs), display many features of circulating cells that are consistent with the original criteria set for an EPC. Basile and Yoder proposed the term pro-angiogenic hematopoietic cells (PACs) to refer to cells that are not in-fact endothelial progenitors but rather “adjuvants” in the process of vascular repair. Our previously identified myeloid angiogenic cells (MACs) would also fit with this definition. Thus, it is likely that different populations of adjuvants cells may exist (see below). The term “EPC” should not be used, hence, to refer PACs/MACs but be restricted to true EPCs (for many researchers, ECFCs).

**Figure 6:**
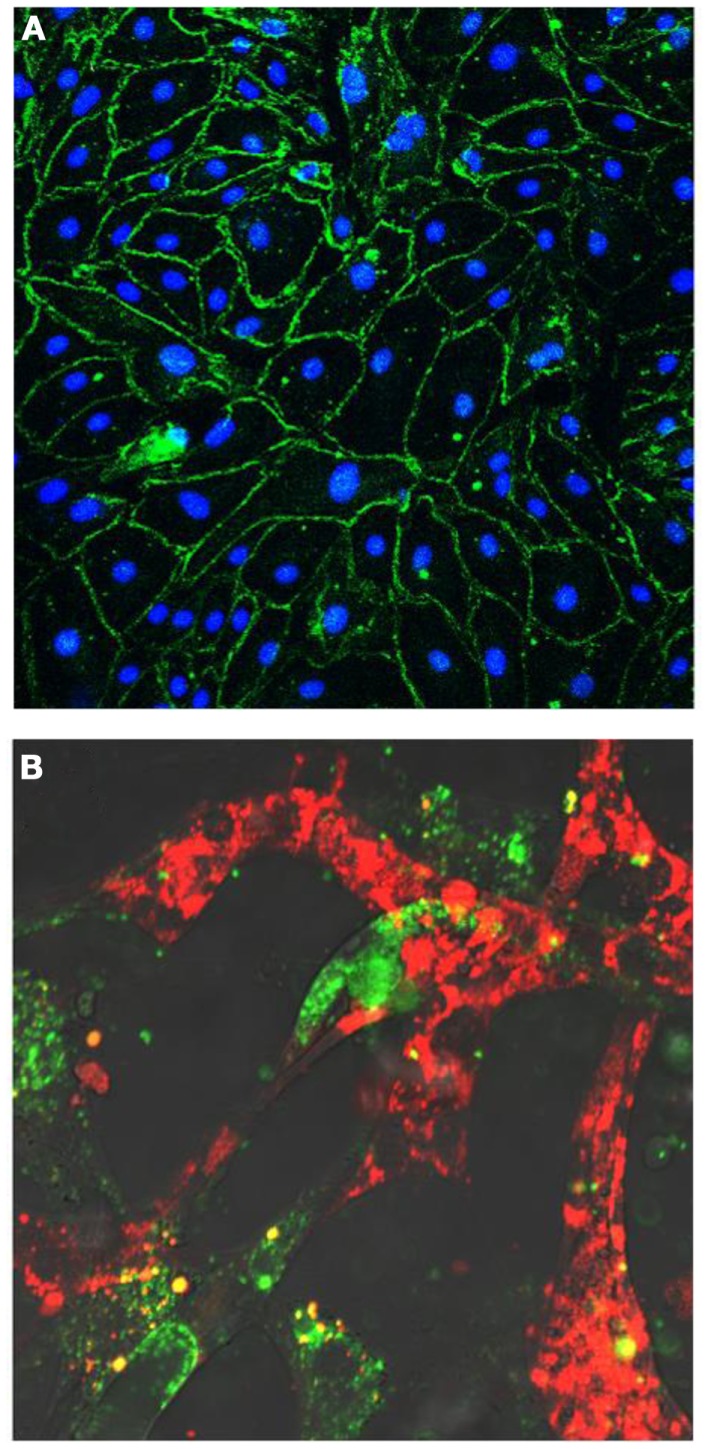
**(A)** Human endothelial colony-forming cells (ECFCs) grow in culture as a cell monolayer and disclose a cobblestone appearance. Cells form tight junctional complexes, shown by Z0-1 staining in green. Nuclei labeled in blue with DAPI. **(B)** Human endothelial colony-forming cells (ECFCs) (labeled in red) form tube-like structures with retinal microvascular endothelial cells (labeled in green) in a 3D Matrigel *in vitro* model.

ECFCs are retrieved with greater efficiency from cord blood compared with peripheral blood; indeed, the former source provides cells with higher proliferation rates and achieves significantly more population doublings than those from peripheral blood (46). A standard method for obtaining ECFCs is by *in vitro* culturing of the mononuclear fraction of blood at high density on Type 1 collagen-coated plates (47). Using this technique and depending on whether they are isolated from cord blood or peripheral blood, ECFCs colonies appear between 2 and 5 weeks and display a characteristic cobblestone-shaped morphology (Figure 6) (47). Research from our group using genome-wide transcriptomics, proteomics, and ultrastructural evaluation has demonstrated ECFCs intrinsic endothelial identity (48, 49). ECFCs have a remarkably high proliferative capacity in comparison with mature endothelial cells and maintain an endothelial phenotype with *ex vivo* long-term expansion (49). ECFCs have robust clonogenic potential, high telomerase activity, and *in vitro* and *in vivo* vessel formation ability (45). Single-cell cloning of ECFCs demonstrates a hierarchic regenerative potential with cells of high proliferative potential (HPP) and low proliferative potential (LPP) similar to what has been observed in hematopoietic stem cells (34, 44). Ingram et al. (44) previously demonstrated that cord blood-derived ECFCs possessed greater HPP (with concomitant enhancement of telomerase activity) than ECFCs isolated from peripheral blood. If ECFCs are to be utilized for regenerative medicine, it may be advantageous to isolate and use an ECFC sub-population with HPP in order to achieve maximum cell number expansion, if required.

ECFCs are positive for a range of endothelial cell markers, including VEGF-R2, VE-cadherin (CD144), CD31, CD105, CD146, and Tie2; negative for hematopoietic cell markers such as CD45, CD14, CD133, CD115, and demonstrate variable positivity to CD34 and CD117 (45, 50). Although evaluation of EPCs has been undertaken widely using a combination of the above cell markers by flow cytometry, as there is no specific antigen for ECFCs, *in vivo* and *in vitro* functional evaluations are required to identify specifically these cells. Thus, studies using only flow cytometry would be determining “putative” EPCs.

In contrast to ECFCs, PACs are CD34^+^CD45^+^CD133^+^CD31^+^ CD14^−^CD235a^−^ (45). Although circulating PACs may localize in a peri-vascular manner at sites of vascular injury, they are not able to integrate in the blood vessels as proper endothelial cells (45). PACs have LPP but they do appear to contribute to vascular repair by paracrine secretion of vasoactive molecules. Similar to PACs, MACs do not populate vessel walls but are pro-angiogenic. MACs are CD45^+^CD14^+^CD68^+^CD163^+^Tie2^−^, CD209^−^CD16^−^ (51). Therefore, as much of the previously conducted work on “EPCs” was undertaken using cells that may not have fit strictly with the above definition of an ECFC and may have been only characterized by flow cytometry, data summarized below should be interpreted with caution.

### Functional properties of EPCs and modulatory mechanisms

Functionally, EPCs present characteristics of endothelial cells (50). Earlier studies emphasized the *in vitro* angiogenic potential of EPCs, their ability to integrate into pre-existing vessels and *de novo* tube formation (32, 43). Furthermore, several research groups, including our own, have demonstrated that EPCs possess *in vivo* potential for direct engraftment, aiding vascular repair and forming well-perfused vasculature in various *in vivo* models (32, 43). For example, the therapeutic potential of EPCs to treat retinal ischemia following intravitreal injection was shown in a mouse model of ischemic retinopathy in which ECFCs homed specifically to ischemic retina and integrated directly within the host vasculature (50). Importantly, these cells assisted in vascular remodeling by forming vascular tubes with subsequent reduction in the area of retinal ischemia and a concomitant increase in normal vasculature (50).

In response to hypoxia, EPCs have the ability to mobilize from their resident niche into the circulating blood and home to ischemic tissues. The mechanisms by which EPCs mobilize and specifically home to areas of ischemia are highly complex and incompletely understood. They involve a range of pathways and stimulatory factors such as EPO (52), VEGF (53), and G-CSF (54) but it also appears that signals released from apoptotic endothelial cells are important for EPC recruitment to sites of vascular damage (55). Since the homing process is hypoxia-mediated, it is unsurprising that HIF-1α appears to be crucial for EPC recruitment to sites of vascular insufficiency (56). On sensing low oxygen levels, HIF-1α induces transcription of SDF-1 and its receptor CXCR4, these known to be essential for EPC migration and adhesion to specific areas of ischemic damage (56). Blocking the CXCR4/SDF-1 signaling pathway inhibits EPC homing and results in their attenuated participation in promoting vascularization (57).

Several factors appear to influence the occurrence and circulating levels of EPCs. These include age (58) and gender (59) with younger women having higher numbers of EPCs. Physical activity also increases numbers of EPCs, especially intense exercise (60). A circadian rhythm on EPC release has been identified with higher levels of EPCs circulating in the morning than in the evening (61). Hypertension, diabetes, smoking habits as well as medications, including statins, angiotensin-converting enzyme inhibitors, and insulin may also affect the production and release of these cells into the circulation (40, 62).

### EPCs in diabetes and diabetic retinopathy

Accumulating evidence supports the concept that both type 1 and type 2 diabetic patients have altered numbers of circulating EPCs (63–65) and, when isolated, show dysfunctional responses including impaired vasoreparative potential and premature senescence (65, 66). Conflicting results, however, have been reported with regard to the levels of circulating EPCs in people with diabetes. Thus, reduced (63), increased (67), or unchanged (68, 69) levels of EPCs compared with those observed in healthy age-matched volunteers have been found in previous studies using flow cytometry techniques. Similarly, although a reduced potential for EPCs to grow in cultures derived from monocytes obtained from peripheral blood was found by some investigators (64, 65, 70), others observed enhanced growth potential when EPCs were isolated from diabetic patients with PDR (67) (see below). Poor glycemic control, as determined by HbA1_c_ levels, appears to be associated with a reduction in numbers of circulating EPCs whereas an adequate control of glycemia seems to increase their numbers (64). Beyond the presence of diabetes, a relatively scarce number of studies have been conducted evaluating EPCs specifically in patients with DR (Table 1) (67–69, 71–74). A summary of these follows.

**Table 1:** **Summary of studies evaluating endothelial progenitor cells in diabetic retinopathy**.

Study	Patients	Controls	Study design	EPCs studied	Results
Lombardo et al. (68)	DM type 2 = 54 - Without angiopathy = 27 - With angiography = 27 (severe NPDR or PDR)	*n* = 24	Cross-sectional	Pre-EPC: CD34^+^/CD133^+^/CD117^+^ EPCs = CD34^+^/CD133^+^/VEGF-R2 Late EPC = CD31^+^/VEGF-R2/CD144^+^	Decreased late-EPCs in DM compared with controls
Zerbini et al. (74)	DM type 1 (*n* = 56) - DM ≥ 23 years no DR = 17 - DM ≤ 7 years no DR = 19 - DM ≤ 20 years NPDR = 20	*n* = 47	Cross-sectional	CD34^+^/CD45^−^/VEGF-R2	No statistically significant difference on EPCs between groups
Brunner et al. (72)	DM type 2 = 126 - With MVD = 66 - Without MVD = 60 - Without DR = 55 - Mild NPDR = 19 - moderate-severe NPDR = 16 - Early PDR = 19 - PDR = 17	none	Cross-sectional	CPCs = CD34^+^/CD133^+^ EPC = CD34^+^/CD133^+^/CD309^+^ Mature EPC = CD34^+^/CD133^+^/CD309^+^/CD31^+^	MVD: Reduced numbers of EPCs with advanced stages of retinopathy No MVD: no statistically significant changes in EPCs and mature EPCs; number of CPCs declined in mild NPDR, increase in PDR, decrease in high-risk PDR
Liu et al. (75)	DM type 2 = 40 - With DR (severe NPDR and PDR) = 20 - With peripheral arterial disease (PAD) = 20	*n* = 20	Cross-sectional	CD34^+^/CD133^+^	Increased EPCs in DR compared with controls
Tan et al. (67)	DM type 1 = 9 and DM type 2 = 13 (undetermined = 1) - With PDR = 23 (~95% had PRP)	*n* = 22	Cross-sectional	CD34^+^/CD45^−^	Increased EPCs in PDR compared with controls
Brunner et al. (71)	DM type 1 = 90 - No DR = 30 - Mild NPDR = 30 - Moderate-severe NPDR = 10 - Early PDR = 10 - High-risk PDR = 10	*n* = 30	Cross-sectional	CPCs = CD34^+^/CD133^+^ EPC = CD34^+^/CD133^+^/CD309^+^Mature EPC = CD34^+^/CD133^+^/CD309^+^/CD31^+^	When compared with diabetics with no DR, EPCs decreased in NPDR EPCs increased in high-risk PDR EPCs decreased in diabetics compared with control group
Fadini et al. (69)	DM type 2 = 60 - No DR = 45 - DR = 15	none	Cross-sectional	EPC = CD34^+^ and CD34^+^/KDR^+^*	Decreased CD34^+^ in DR. (No decrease in CD34^+^/KDR^+^ in DR detected)
Lee et al. (73)	DM type 2 = 45 - No DR = 15 - NPDR = 15 - PDR = 15	*n* = 15	Cross-sectional	CD34^+^	Increased CD34^+^ in DM compared with control. Increased CD34^+^ with increasing severity of DR (no DR–NPDR–PDR)

*DM, diabetes mellitus; DR, diabetic retinopathy; NPDR, non-proliferative diabetic retinopathy; PDR, proliferative diabetic retinopathy; EPC, endothelial progenitor cell; CFU, colony-forming unit; MVD, macrovascular disease*.

*Both CD34^+^ and CD34^+^/KDR^+^ were measured.

#### Clinical studies on EPCs and DR

In a cross-sectional study including 60 type 2 diabetic patients with and without peripheral arterial disease and with and without severe NPDR and PDR (DR^−^/PAD^−^ *n* = 15, DR^−^/PAD^+^ *n* = 30, DR^+^/PAD^−^ *n* = 5, and DR^+^/PAD^+^ *n* = 10), Fadini and collaborators (69) identified reduced levels of CD34^+^ cells in patients with DR when compared with those without it. No differences in levels of CD34^+^/KDR^+^ (KDR = VEGF-R2) cells between these groups were, however, found. Patients with peripheral arterial disease had reduced levels of CD34^+^/KDR^+^ cells.

Lee and colleagues (73) evaluated, in a cross-sectional study, the concentration of circulating EPCs, defined as CD34^+^ cells or c-Kit^+^ cells, in a group of 45 type 2 diabetic patients and compared them with those observed in 15 age- and gender-matched control subjects. Patients were classified as having no DR (*n* = 15), NPDR (*n* = 15), or PDR (*n* = 15). Circulating CD34^+^ cell numbers were higher in diabetic patients compared with control subjects and increase with increasing staging of the disease (PDR > NPDR > no DR).

Brunner and collaborators (71) undertook a case–control cross-sectional study, which included 90 patients with type 1 DM with no DR (*n* = 30), mild NPDR (*n* = 30), moderate to severe NPDR (*n* = 10), mild to moderate PDR (*n* = 10), and high-risk PDR (*n* = 10). The study included an age-, gender-, and body-mass index-matched control group (*n* = 30). CD34^+^/CD133^+^ (which they labeled “circulating progenitor cells” or “CPCs”), CD34^+^/CD133^+^/CD309^+^ (labeled as “EPCs”), and CD34^+^/CD133^+^/CD309^+^/CD31^+^ (labeled as “mature EPCs” of “mat-EPCs”) were measured. When compared with diabetic patients without DR, levels of CPCs, EPCs, and mat-EPCs were found to be reduced in NPDR; EPCs and mat-EPCs were found to be increased in PDR. EPC and mat-EPC number was reduced in patients with diabetes when compared with healthy subjects. As a more recent follow-up, this group expanded their observations to a group of 126 patients with type 2 diabetes, with or without macrovascular disease and with no DR (*n* = 55), mild NPDR (*n* = 19), moderate to severe NPDR (*n* = 16), early PDR (*n* = 19), and high-risk PDR (*n* = 17) (72). In contrast to their previous findings, they failed to detect statistically significant differences in EPC numbers in patients with type 2 diabetes without macrovascular disease at different stages of DR. However, in patients with macrovascular disease, EPCs declined with advancing stages of retinopathy.

Liu and colleagues (75) examined type 2 diabetic patients with DR (severe NPDR and PDR, *n* = 20), type 2 diabetic patients with peripheral arterial disease (*n* = 20), and healthy age- and gender-matched volunteers (control group, *n* = 20) and found increased levels of circulating EPCs in patients with DR when compared with the control group. EPCs were defined as CD133^+^/CD34 ^+^ cells, as determined by flow cytometry. An increase in early EPC-CFU (colony-forming units) count was also observed in patients with DR. Serum levels of nerve growth factor (NGF) and brain-derived neurotrophic factor (BDNF) were found to be increased in patients with DR and correlated with levels of circulating EPCs.

Tan and collaborators (67) undertook a study on a group of 23 diabetic patients, type 1 (*n* = 9) and type 2 (*n* = 13) (*n* = 1 not determined) with PDR and 22 healthy controls. The great majority of patients (95%) had received PRP. They found that the number of circulating ECFCs, determined as CD34^+^/CD45^−^ cells by flow cytometry, was increased in PDR. Furthermore, mononuclear cells obtained from patients who had PDR were more likely to grow ECFC colonies in culture than those obtained from healthy controls. ECFCs from patients with PDR demonstrated reduced migration (PDR *n* = 2) and reduced incorporation into vascular tubes (PDR *n* = 3) *in vitro*. Microarray analysis of ECFCs from patients with PDR (*n* = 2) demonstrated up-regulation of thrombospondin-1 and tissue inhibitor of matrix metalloproteinases-3 (TIMP-3); western blotting confirmed increased levels of both proteins in ECFC lysates.

Lombardo and associates (68) did not find a significant reduction in the number of EPCs (CD34^+^/CD133^+^/VEGF-R2^+^) in patients with type 2 diabetes when compared with those from a group of healthy individuals (control group). Subsets of patients with diabetes with or without angiopathy were found to have significantly higher numbers of what was termed pre-EPCs (CD34^+^/CD133^+^/CD117^+^). However, numbers of CD31^+^/VEGF-R2^+^/VE-cadherin^+^ cells (CD144^+^), termed by the authors “late-EPCs,” were reduced in patients with diabetes type 2 recently diagnosed (within 1 year of diagnosis) with no clinical evidence of angiopathy (*n* = 27) and in those with peripheral arterial occlusive disease and DR (severe NPDR or PDR) (*n* = 27) when compared with the control group (*n* = 24). The number of circulating endothelial cells, matured endothelial cells thought to sloughed off from the vascular intima, where increased in diabetic patients.

Zerbini and colleagues (74) evaluated three groups of type 1 diabetics, with long-standing (≥23 years; *n* = 17) and short-standing (≤7 years; *n* = 19) diabetes and no retinopathy and with NPDR (diabetes of ≤20 years standing; *n* = 20). A group of age- and gender-matched healthy volunteers were also included for each of the above (with *n* = 17, 12, and 18 subjects, respectively). No differences were found in levels of CD45^−^/CD34^+^/VEGF-R2^+^ cells between the above different groups studied. However, the number of colonies formed in the Hill assay was increased in patients with NPDR when compared with those in the control group. Patients with milder retinopathy tended to form more colonies than patients with more severe retinopathy. In patients with long-standing diabetes and no retinopathy, the colony counts were similar to those in control subjects.

The interpretation of the previously presented findings is challenging. For instance, both high (67, 71) and low (69, 72) levels of circulating EPCs have been reported in patients with severe NPDR and PDR when compared with those in individuals with no or mild retinopathy and with healthy subjects without DM. It is possible that discrepancies observed may relate to methodological differences among studies, specifically, and as underlined above, the different criteria used to define an EPC. Other factors need to be taken into consideration. Some studies included small number of subjects (67–69, 73–75), especially when grouped in the different disease progression categories (68, 71–75), which may have had an effect on the statistical evaluation of the data (a sample power calculation was not given in any of the above studies). All published studies on the subject have been cross-sectional (67–69, 74, 75) and, subsequently, truly unable to evaluate the influence of changes in EPCs in relation to disease progression. Few addressed the relationship between number/function of EPCs and severity of DR (71–73) and none attempted to relate them to the presence/extension of retinal ischemia, as determined by using imaging modalities such as fluorescein angiography. As retinal ischemia would seem to be a main driver for eliciting a vasoregenerative response led by EPCs, correlating the latter with retinal ischemia would seem fundamental. In this regard, prospective longitudinal studies from our own laboratory are underway to address the relationship between retinal ischemia and the EPC response. As information with regard to flow cytometry techniques used in these studies was limited, variability in methodological aspects of flow cytometry could have also accounted for inconsistencies in the results observed.

## Potential Use of EPCs in Diabetic Retinopathy

Endothelial progenitor cells could be potentially used in the management of patients with DR with two different purposes: (1) as cell biomarkers or “prognosticators” and (2) as a potential therapy.

### EPCs as biomarkers of disease severity

Endothelial progenitor cells have been proposed as useful biomarker for cardiovascular events and cancer progression (76, 77). In a similar manner, it is possible that EPC number/function could be used to predict which individuals may be at higher risk of losing sight from DME or PDR. Prospective adequately powered cohort studies would be needed to elucidate the potential use of EPCs as cell biomarkers for DME and PDR and take into account other risk factors and variables known to potentially affect retinopathy. It is possible that other cells besides those strictly defined as EPCs (for many, ECFCs) may be used for this purpose. As identification of ECFCs in culture requires a period of around 4–6 weeks, this strategy may be less practical and appealing than evaluating levels of other circulating cell types by flow cytometry. Research in this area could prove fruitful as predicting individuals at higher risk of visual loss would be extremely helpful to the currently overstretched DR screening programs and health care systems. This would allow a personalized follow-up strategy based on the individual’s risk of progression and would avoid close follow-up with the benefit to patients and health care systems.

### EPCs as a potential therapy to treat DR

The potential use of EPCs to treat DR is exciting but extensive work is required before they could be introduced in clinical practice. Firstly, the type of cell(s) to be used and the number of cells required should be determined. If a cell, alone, were to be used for therapeutic purposes, those with the highest reparative potential (i.e., ECFCs) may be chosen. Although not the subject of this review, pericyte progenitor cells (PPCs) have been recently recognized (78, 79). As both, endothelial cells and pericytes are affected in DR, it would seem reasonable to consider the possible administration of both progenitors in order to treat this vascular disorder. Recently, Lee and co-workers (80) demonstrated that CD34^−^ cells may modulate the inherent characteristics and behavior of ECFCs. They showed *in vitro* that ECFCs arising from cultures in which CD34^+^ and CD34^−^ cells are present (to which they referred to as “hybrid ECFCs”) have higher proliferation capacity and slower senescence than those ECFCs grown in cultures lacking CD34^−^ cells (which they referred to as stem-ECFCs). The later, however and importantly, demonstrated higher endothelial cell differentiation. Both cell types (hybrid ECFCs and stem-ECFCs), expressed ECFC surface markers, including CD133, VEGF-R2, CXCR4, and c-kit) but did not expressed hematopoietic lineage markers (CD11b, CD14, CD45). Before CD34^−^ cells are used to condition ECFCs prior to treatment (80) it would be important to determine that these ECFCs do not have an increased ability to form “neovessels.” Whereas in other organs “neovascularization” may be beneficial, for instance in the heart, this would not be the case in the retina where “revascularization” or “healing” of pre-existing blood vessels rather than neovascularization should be sought. Other cells may be also considered. As PACs are likely to act as adjuvants in the process of “vascular regeneration,” further consideration to administering these, in addition, should be given. However, the possibility for PACs to develop into pro-inflammatory cells, which could have potentially a deleterious effect in the retina, should be cautiously balanced.

Secondly, the number of cells required would need to be estimated. It is likely that it would depend on the route of administration selected as well as on the stage of disease at which patients are planned to be treated. The route of administration will need to be carefully chosen. Local delivery into the eye by means of intravitreal injections would be an option, as many clinical trials (81–84) using anti-VEGF therapy (Ranibizumab, Bevacizumab, and Aflibercept) have demonstrated their safety even when administered monthly. However, considering the fact that DR often affects similarly both eyes and that DM is a systemic disorder in which other organs besides the eye are affected and may benefit from this therapy, systemic administration should not be ruled out. Systemic administration, if safe, could be given repeatedly, if required, and at an early stage of disease.

Thirdly, the population most likely to benefit from treatment with EPCs should be defined. EPCs could be used in an attempt to re-vascularize areas of retinal ischemia in patients who have already developed retinal vessel dropout as determined by fluorescein angiography. They could be used also to improve retinal vessel function in patients with increase vessel permeability and subsequent DME with or without retinal ischemia being present. If the balance between beneficial effects and side effects were to be appropriate, EPCs could be used even at very early stages of retinopathy, before marked retinal vessel abnormalities occurred in an attempt to abort progression to sight threatening complications. Alternatively, maneuvers aimed at increasing levels of circulating endogenous EPCs, by stimulation of the bone marrow and other EPC niches, and their homing to sites where needed (i.e., retina) would be an appealing option.

Fourthly, the most appropriate outcome measures that would allow treatment effects to be determined in clinical trials should be sought. For instance, if ECFCs adequately incorporate into damaged retinal blood vessels in ischemic retina oxygen levels in retinal veins may be expected to drop, as it has been shown that there is increased oxygen saturation in people with DR (85, 86), which may relate, at least partially, by the poor distribution of blood to retinal tissue result of capillary dropout. Fluorescein angiography and, in the future, when fully developed for clinical use, differential phase-contrast swept-source OCT may be able to provide adequate information with regard to ECFCs incorporation to retinal vessel walls by demonstrating the presence of capillaries in retinal areas were prior to treatment were not present and, in the case of the former, by demonstrating improve circulation in previously ischemic areas. Retinal functional studies, such as multifocal ERG responses, may be able to determine improved responses in those areas where revascularization takes place.

Lastly but importantly, potential side effects of the treatment will need to be carefully evaluated. Data from available completed clinical trials, however, suggests that ECFCs should be safe for clinical use.

## Conclusion

Available data suggests EPCs are essential on maintaining retinal vessel integrity and homeostasis. While some patients seem to have inherent regenerative capacity, the molecular and cellular basis for this has not been demonstrated. It seems likely that high levels of reparative cells could underpin less risk of DR progression. However, the precise role of EPCs in DR remains to be determined. Experimental and adequately powered prospective cohort clinical studies are required to better understand the possible role of EPCs in the occurrence and progression of this disease. The possibility of using EPCs as cell biomarkers of the sight threatening complications of DR, namely DME and PDR and as cell therapy is an exciting one. Given the complexity of DM and DR, with multiple factors modulating disease progression, a joint effort by multidisciplinary research teams is likely to be needed in order to achieve this goal.

## Conflict of Interest Statement

The authors declare that the research was conducted in the absence of any commercial or financial relationships that could be construed as a potential conflict of interest.
